# Two-headed mutants of the lamprey, a basal vertebrate

**DOI:** 10.1186/s40851-016-0058-z

**Published:** 2016-11-16

**Authors:** Daichi G. Suzuki

**Affiliations:** 1 Graduate School of Life and Environmental Sciences, University of Tsukuba, 1-1-1 Tennodai, Tsukuba, Ibaraki 305-8572 Japan; 2 Nobel Institute for Neurophysiology, Department of Neuroscience, Karolinska Institutet, SE-17177, Stockholm, Sweden

**Keywords:** Lamprey, Teratology, Two-headed twin, Axial duplicity, Bicephaly

## Abstract

**Background:**

This is the first report of two-headed (bicephaly) lamprey twins. Although lampreys sit at a crucial phylogenetic position, there are only a few reports on their teratology and developmental abnormalities.

**Results:**

Two-headed mutants were obtained by artificial fertilization in the laboratory as spontaneous occurrences. All mutants were derived from single fertilizations using single male and female gametes, suggestive of a genetic background. The anterio-posterior position of the axonal bifurcation and symmetricity varied in each mutant. Other malformations were coincidently observed, including pericardial edema, yolk sac edema and axial bending. Asymmetrical (lateral- branched) mutants displayed more severe abnormalities in the cranial nerves than symmetrical mutants.

**Conclusion:**

Two-headed mutants of the lamprey are described. These mutants have similar malformations to dorsal blastopore lip-transplanted lamprey embryos, suggesting that they could be generated by a disorder in head-organizing activity.

**Electronic supplementary material:**

The online version of this article (doi:10.1186/s40851-016-0058-z) contains supplementary material, which is available to authorized users.

## Background

Malformed mutants, or so-called “monsters”, have attracted the attention of morphologists since the inception of the discipline. For example, Étienne Geoffroy Saint-Hilaire and his colleagues, including his son Isidore, described many developmental anomalies and sought to explain the mechanisms underlying their production [1, 2]. As described in their works, conjoined twins, referred to as “axial duplicity” or “double monsters” in Bateson [3], also represent striking examples of such anomalies.

More recently, in the field of evolutionary developmental biology, researchers have focused more on malformations, as these can represent developmental constraint, variability, and evolvability [4–6]. In fact, axial bifurcation in the caudal fin of the twin-tail goldfish is caused by ventralization during early embryonic development, which is in turn due to a mutation in a *chordin* gene that may have occurred during domestication [7]. Developmental malformations in lampreys, which belong to a basal group of vertebrates (cyclostomes), may similarly provide new insights into the early evolution of vertebrates. However, only a few reports on lamprey teratology and developmental abnormalities have been published to date (e.g., [8–10]).

Here I report two-headed conjoined (bicephaly) mutants in the Arctic lamprey, *Lethenteron camtschaticum*, which were unexpectedly obtained following artificial fertilization in the laboratory. These mutants have similar malformations to dorsal blastopore lip-transplanted embryos [11], suggesting that they may be generated by a disorder in head-organizing activity.

## Methods

Adult lampreys *(Lethenteron camtschaticum*) were collected from the Shiribeshi-Toshibetsu River, Hokkaido, Japan in 2012. In the next spawning season (May to June 2013), the animals were anesthetized in ethyl,3-aminobenzoate methanesulfonate (MS-222). Mature eggs and sperm were squeezed from adults and fertilized in vitro. Embryos were cultured at 16 °C, fixed in 4% paraformaldehyde in 0.1 M phosphate-buffered saline (PBS) overnight, dehydrated in a graded methanol series, and stored in 100% methanol at −20 °C.

Whole-mount immunofluorescence with anti-acetylated tubulin (Sigma, T6793) antibodies was performed according to the protocol described by Kuratani et al. [12] with some minor modifications. Briefly, fixed embryos stored in methanol were washed in TBST containing 5% dimethylsulfoxide (TSTd). The embryos were then blocked with 5% non-fat dry milk in TSTd (TSTM). They were incubated with the primary antibody (diluted 1:1000 in TSTM) for 2–4 days at room temperature. After washing with TSTd, samples were incubated with a secondary antibody (Invitrogen, Alexa fluor 555, A21424) diluted 1:200 in TSTM. Then embryos are washed by TSTd several times, treated RNase and counter-stained by YOYO-1. After a final wash in TSTd, embryos were dehydrated and clarified in a 1:2 mixture of benzyl alcohol and benzyl benzoate (BABB) and then examined using a confocal laser microscope (LSM 510, Zeiss).

Paraffin sections were cut at a thickness of 8 μm and stained with hematoxylin and eosin, according to a standard protocol.

## Results and discussion

**Fig. 1:**
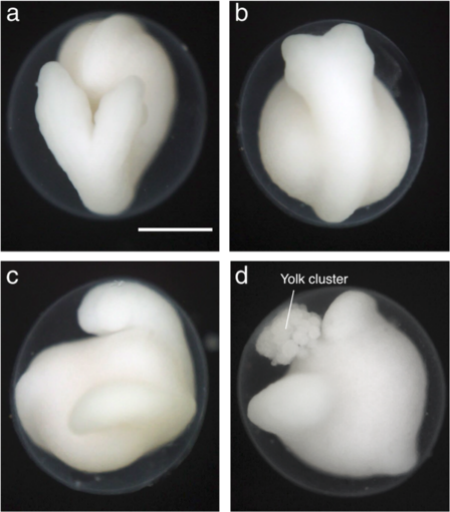
Two-headed lamprey embryos. **a**-**d** Pre-hatching embryos at the same magnification. The antero-posterior position of the axonal bifurcation and symmetricity varied among mutants. The exo-embryonic yolk cluster can be seen in (**d**). Scale bar: 500 μm

I obtained two-headed embryos from one female lamprey by artificial fertilization (at least 25 embryos). As *L. camtschaticum* females spawn large numbers of eggs (tens of thousands [13]) and dead embryos were immediately removed to keep the batch clean, I could not specify how many mutants were generated and calculate the malformation rate precisely. However, it is clear that the occurrence of two-headed mutants is very rare. The antero-posterior position of the axonal bifurcation and symmetricity varied among mutants (e.g., Fig. 1a–d). Some embryos had exo-embryonic yolk clusters (Fig. 1d), as is often the case with normal lamprey embryos. Over the 10 years that lamprey embryos have been collected in this laboratory, two-headed mutants have only been obtained from one female, even though sperm from the same male was used to fertilize eggs from other females and the same fertilization/incubation conditions have always been used. Although some environmental effect to the female individual could cause maternal effects and the two-headed mutation, migrant adults collected upstream were exposed to the same condition during their handling and maintenance process, without exhibiting similar mutations. Therefore, I infer that the malformation may be attributable not to an artificial or environmental effect, but to some natural (spontaneous) occurrence in the genetic background, similar to those observed in other vertebrates, including humans [3, 14–16]. This is the first known report of two-headed lamprey twins, although complete twin embryos have been reported previously [10]. It would be difficult to observe these lamprey mutants in the wild, as they die quickly and, even if they manage to survive, tend to hide in the sandy bottom.

**Fig. 2:**
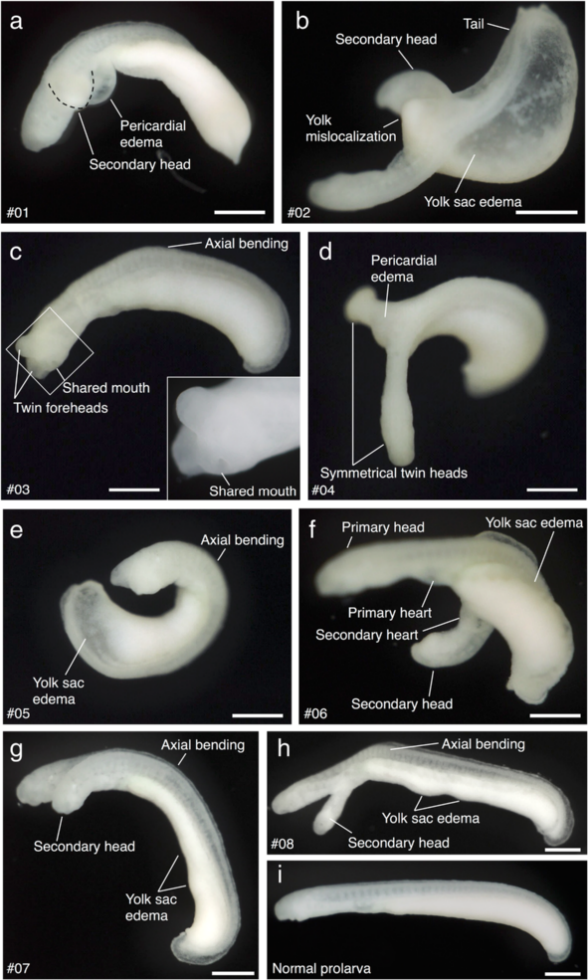
Two-headed lamprey prolarvae. **a**-**h** Two-headed mutants after hatching. Identity numbers for each mutants are shown as #01–08. Mutants show various types of defects. For a summary of malformations, see Additional file 1. **i** A normal prolarva (st. 26). Scale bars: 500 μm

During incubation, some two-headed embryos (particularly severely malformed mutants) died before hatching. I obtained 21 mutants (identity numbers are provided as #01–#21, see also Additional file 1) after hatching (e.g., Fig. 2a–h), which were nearly the same size as normal prolarvae (Fig. 2i). There were two general types of malformation: lateral and symmetrical bifurcation. Laterally bifurcated mutants possessed secondary head, which was smaller than primary head, while symmetrically bifurcated mutants showed no such significant difference between the two heads. Depending on the antero-posterior position of the axonal bifurcation, they showed various types of defects, for example, a shared mouth (Fig. 2c) and double hearts (Fig. 2d). While normal prolarvae usually show rhythmic neck movements, most of the mutants moved non-rhythmically, suggesting that twin heads “compete” with each other for motor control. In addition, other malformations were coincidently observed, including pericardial edema (Fig. 2a, d, 24% of obtained mutants), yolk sac edema (Fig. 2b, e–h, 48%) and axial bending (Fig. 2e–g, 57%). This suggests that the causal factors of these malformations are linked and that one malformation tends to involve others (for the sample list with summary of malformations in each mutants, see Additional file 1).

**Fig. 3:**
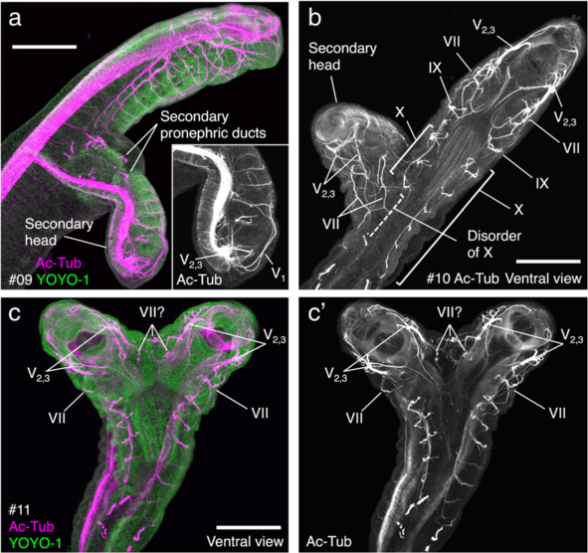
Immunofluorescence analysis. Identity numbers for each mutants are shown as #09–11. **a**, **b** Lateral bifurcated mutants. **c** A symmetrically bifurcated mutant. Nerve fibers and pronephric ducts are visualized by anti-acetylated tubulin antibody in magenta and cell nuclei are labeled by YOYO-1 in green in (**a**) and (**c**). In (**b**), (**c**’) and the inset in (**a**), only the signal of anti-acetylated tubulin antibody is shown. Scale bars: 200 μm

To survey the morphological effects of the malformation on peripheral nerves, I performed immunofluorescence using an anti-acetylated tubulin antibody (for the normal development of the lamprey peripheral nerve, see [12]). The lateral-branched twins had a malformed cranial nerve in the secondary head, which was more severe than that seen in symmetrically bifurcated twins (Fig. 3a, b). Some lacked mouth openings (Fig. 3a), and cranial nerve fibers were entangled (e.g., ophthalmicus profundus nerve [V_1_] in Fig. 3a) or fused in the ventral midline (e.g., maxillomandibular [V_2,3_] and facial [VII] nerves in Fig. 3b). This may be due to a restricted cell source due to the asymmetrical bifurcation, resulting in cranial malformation and axon misguidance. In addition, peripheral nerves in the primary axis were also disordered because of the bifurcation (e.g., vagus nerve [X] in Fig. 3b). Also, as shown in Fig. 3a, secondary pronephric ducts were observed immediately adjacent to the heart, consistent with normal heart development in the lamprey [17]. As the nephric system is derived from intermediate mesoderm, which arises between the somite and lateral plate mesoderm of the trunk [18], this observation supports the idea that the heart is formed in the rostral-most trunk region in the lamprey.

**Fig. 4:**
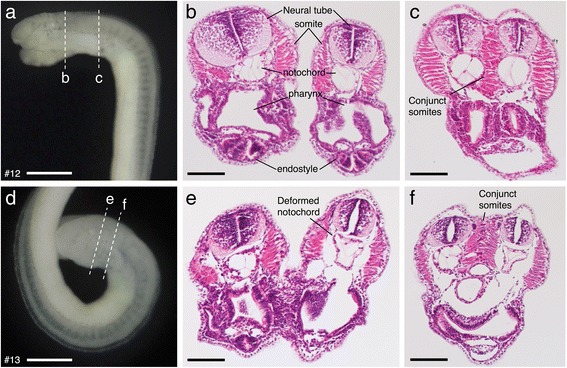
Histological analysis for symmetrically bifurcated mutants by hematoxylin-eosin (HE) staining. Identity numbers for each mutants are shown as #12 and #13. **a**, **d** Lateral view of the mutants. Sectioned levels are shown. **b**, **e** Transversal sections of two-headed region. **c**, **f** Transversal sections of conjunct region. Scale bars: 500 μm for **a**, **d**, 100 μm for (**b**, **c**, **e**, **f**)

In the symmetrical two-headed twins, cranial nerves were entangled in the conjoined regions (e.g., putative facial nerve fibers [VII?] in Fig. 3c). In these regions, molecular cues for axon guidance may be provided from both sides of the bifurcated axes, resulting in the disruption of cranial innervation. I performed histological analysis to survey other morphological effects of the malformation (Fig. 4). In symmetrical two-headed twins, most organs are symmetrically present in both primary and secondary axes (see Fig. 4b), although some organs (e.g., the notochord in Fig. 4e) were morphologically deformed. In the bifurcating region, muscle fibers from primary and secondary axes meshed with each other in the same somite (Fig 4c, f), indicating that the bifurcation could occur *within* the somite (i.e., not *between* somites).

The mutants described in this study are similar to the experimentally induced conjoined lamprey twins, produced through dorsal blastopore lip transplantation, reported by Yamada [11]. The monumental work of Spemann and Mangold [19] demonstrated that transplantation of the dorsal blastopore lip in amphibians could induce conjoined twin embryos and this region acts as the head organizer. Although the developmental mechanisms for the head-organizer in lampreys are not known in detail, it is possible that the two-headed mutants described in this study were generated by some disruption of the head-organizing activity. In *Xenopus*, Siamois (Sia) and Twin (Twn) induce expression of head-organizer-specific genes, such as *Goosecoid* (*Gsc*), and double knockdown of Sia and Twn results in two-headed malformations [20]. However, Sia and Twn orthologs are absent in non-amphibian vertebrates, suggesting that there is species-specific diversity in the developmental mechanisms of head-organizer activity [20]. To investigate the degree to which these mechanisms are conserved or diversified in vertebrates, and to reveal the evolutionary origin of the vertebrate head, further comparative studies on the vertebrate head-organizer are needed.

## Conclusions

I report two-headed mutants in a lamprey, obtained from artificial fertilization using single male and female gametes, suggestive of a genetic background. These mutants have similar malformations to dorsal blastopore lip-transplanted lamprey embryos, suggesting that they may be generated by a defect in head-organizing activity.

## Additional file

Additional file 1: The sample list with summary of malformations. (XLS 27 kb)

## Abbreviations

V_1_: Ophthalmicus profundus nerve
V_2,3_: Maxillomandibular nerve
VII: Facial nerve
X: Vagus nerve
XI: Glossopharyngeal nerve
